# Overexpression of stathmin in oral squamous-cell carcinoma: correlation with tumour progression and poor prognosis

**DOI:** 10.1038/sj.bjc.6602991

**Published:** 2006-02-21

**Authors:** Y Kouzu, K Uzawa, H Koike, K Saito, D Nakashima, M Higo, Y Endo, A Kasamatsu, M Shiiba, H Bukawa, H Yokoe, H Tanzawa

**Affiliations:** 1 Department of Clinical Molecular Biology, Graduate School of Medicine, Chiba University, 1-8-1 Inohana, Chuo-ku, Chiba 260-8670, Japan; 2 Division of Dentistry and Oral-Maxillofacial Surgery, Chiba University Hospital, 1-8-1 Inohana, Chuo-ku, Chiba 260-8670, Japan; 3 Center of Excellence (COE) Program in The 21st Century, Graduate School of Medicine, Chiba University, 1-8-1 Inohana, Chuo-ku, Chiba 260-8670, Japan

**Keywords:** oral squamous-cell carcinoma, stathmin, proteomics, biomarker, poor prognosis

## Abstract

Stathmin is an intracellular phosphoprotein that is overexpressed in a number of human malignancies. Our previous study using proteomic profiling showed that significant upregulation of stathmin occurs in oral squamous-cell carcinoma (OSCC)-derived cell lines. In the current study, to determine the potential involvement of stathmin in OSCC, we evaluated the state of stathmin protein and mRNA expression in OSCC-derived cell lines and human primary OSCCs. A significant increase in stathmin expression was observed in all OSCC-derived cell lines examined compared to human normal oral keratinocytes. In immunohistochemistry, 65% of the OSCCs were positive for stathmin, and no immunoreaction was observed in corresponding normal tissues. Real-time quantitative reverse transcriptase–polymerase chain reaction data were consistent with the protein expression status. Moreover, stathmin expression status was correlated with the TNM stage grading. Furthermore, we found a statistical correlation between the protein expression status and disease-free survival (*P*=0.029). These results suggest that expression of stathmin could contribute to cancer progression/prognosis, and that stathmin may have potential as a biomarker and a therapeutic target for OSCC.

Oral squamous-cell carcinoma (OSCC) is the most common cancer of the head and neck and accounts for over 300 000 new cancer cases worldwide annually (Lippman *et al*, 2005). With the currently available clinical assessment and treatment methods, patients are often diagnosed at late stages and the survival rate has not improved substantially. This highlights the need for continued efforts to discover suitable biomarkers for early diagnosis of the disease and to understand the disease pathogenesis as a first step toward improving treatment. Elucidation of the genetic changes leading to the development of OSCCs will probably result in improved molecular assays for the early diagnosis of, therapy for, and improved prognosis of this cancer. The availability of biomarkers of malignancy would also be key for monitoring cancer recurrence and evaluating the efficacy of novel treatment or chemopreventive agents.

The proteomic study includes post-translational modifications such as acetylation, ubiquitination, phosphorylation, or glycosylation (Larsson *et al*, 2000; Charlwood *et al*, 2001). Proteomic methods can detect the functioning units of expressed genes using protein fingerprinting (Wilkins *et al*, 1996; Pandey and Mann, 2000). In addition, many cancer biomarkers are a manifestation of differences in post-transcriptional splicing, post-translational modifications, or both. Thus, proteomic tools are used increasingly in the post-genomic era to discover new cancer biomarkers. Such information will likely prove to be crucial in cancer prognosis, diagnosis, prevention, and therapy, with the ultimate goals being therapeutic target discovery, rational drug design, and identification of early-detection surrogate biomarkers (Pandey and Mann, 2000; Rudert, 2000). However, comprehensive proteomic study of OSCCs to identify the targets of molecules has not yet been undertaken. In our previous study, using a fluorescent two-dimensional differential in-gel electrophoresis (2-D-DIGE) system and matrix-assisted laser desorption/ionisation time-of-flight mass spectrometry (MALDI–TOF/MS), we compared protein expression profiles in human normal oral keratinocytes (HNOKs) and OSCC-derived cell lines and identified several OSCC-associated proteins (Koike *et al*, 2005). Among them, the protein stathmin was associated with tumours. At present, it is unclear whether stathmin is associated with oral carcinogenesis. The major purpose of this study was therefore to examine the expression of stathmin protein and mRNA in OSCC-derived cell lines and human primary OSCCs.

## MATERIALS AND METHODS

### Tissue specimens and cell lines

Tissue samples from 81 unrelated Japanese patients with primary SCC of the oral cavity who were treated at the Chiba University Hospital were obtained at the time of surgical resection between 1998 and 2005. Tumours and patient-matched normal epithelium were obtained at the time of surgery at Chiba University Hospital after the patients' informed consent was obtained under a protocol reviewed and approved by the institutional review board of Chiba University. The respected tissues were divided into two parts, one of which was frozen immediately after careful removal of the surrounding normal tissues and stored at −80°C until protein isolation; the second part was fixed in 10% buffered formaldehyde solution for pathologic diagnosis and immunohistochemical staining. Histopathologic diagnosis of each neoplastic tissue was performed according to the World Health Organization criteria by the Department of Pathology, Chiba University Hospital. Clinicopathologic staging was determined by the TNM classification of the International Union against Cancer. All patients had SCC that was confirmed histologically, and tumour samples were checked to ensure that tumour tissue was present in more than 80% of the specimens.

The follow-up data have been collected until November 2005 or until the patients' death, and the occurrence of metastasis and/or local recurrence was recorded. For the analyses, the patients were divided into the following two groups depending on the different kind of outcome. Patients who had no evidence of disease (local recurrence or metastasis) during follow-up were regarded as a good prognosis group. In contrast, patients who developed local recurrence, distant metastasis, and/or death during follow-up, whichever occurred first were regard as a poor prognosis group. A follow-up was available for all patients, ranging from 3 to 60 mouths after the primary treatment (median follow-up, 30.3 months). The OSCC-derived cell lines used in this study were HSC-2, HSC-3, HSC-4, Ca9–22 (Human Science Research Resources Bank, Osaka, Japan), OK92 (established from carcinoma of the tongue in our department) (Takahashi *et al*, 1989), and Sa3 (provided by Dr Fujita at Wakayama Medical University, Wakayama, Japan). All OSCC-derived cell lines were grown in Dulbecco's modified Eagle medium/F-12 HAM (Sigma-Aldrich Co., St Louis, MO, USA) supplemented with 10% fetal bovine serum (Sigma) and 50 U ml^−1^ penicillin and streptomycin (Sigma). Healthy oral gingival specimens were collected from 22- to 35-year-old patients at Chiba University Hospital. The institutional review board of Chiba University approved all relevant protocols. Five independent HNOK cell lines were cultured and maintained in defined keratinocyte-SFM (Gibco BRL, Gaithusberg, Germany) (Koike *et al*, 2005).

### Protein and mRNA extraction

Protein was extracted from the cells when they reached 80–90% confluence; they were washed twice with phosphate-buffered saline (PBS), scraped into a tube, and centrifuged briefly. The cell pellets were incubated for 30 min in a lysis buffer (LB) containing 7 M urea, 2 M thiourea, 4% w v^−1^ CHAPS, and 10 mM Tris pH 8.0, and lysed by sonication (3 × 10 s pulses on ice). The sample was centrifuged at 13 000 r.p.m. for 20 min. The supernatant containing the cell proteins then was recovered, and the protein concentration was measured with a Protein Assay Kit (Bio-Rad Laboratories, Hercules, CA, USA) and adjusted to 1 mg ml^−1^ with LB. The pH of the protein sample was adjusted to 8.5 with 30 mM Tris-HCl. Total RNA was extracted using Trizol Reagent (Invitrogen Life Technologies, Carlsbad, CA, USA), according to the manufacturer's instructions. Each specimen of extracted RNA was stored separately at −80°C until use.

### Immunofluorescence

To examine the protein expression of stathmin, we performed immunofluorescence analysis. Briefly, the cells were washed with PBS and fixed in 4% paraformaldehyde for 10 min at 37°C followed by absolute methanol for 10 min at 4°C and blocked in PBS containing 1% skimmed milk for 10 min. The samples then were incubated with affinity-purified rabbit antihuman stathmin polyclonal antibody (Abcam Inc., Cambridge, CA, USA) at a dilution of 1 : 100 for 2 h, rinsed twice with PBS, and incubated with goat anti-rabbit secondary antibody labelled with Alexa Fluor 350 (Molecular Probes, Leiden, the Netherlands) for 1 h. The samples were observed under a Leica TCS2-MP confocal system (Leica Laserteknik, Mannheim, Germany) and Coherent Mira tunable pulsed titanium sapphire laser (Coherent Laser Group, Santa Clara, CA, USA) (Kasamatsu *et al*, 2005).

### Western blot analysis

Protein extracts were electrophoresed on 11% sodium dodecyl sulfate–polyacrylamide gel electrophoresis gels, transferred onto PVDF membranes (Bio-Rad), and blocked for 1 h at room temperature in 5% skim milk. Immunoblot PVDF membranes were washed with 0.1% Tween-20 in TBS (TBS-T) five times, and 2 *μ*g ml^−1^ affinity-purified rabbit antihuman stathmin polyclonal antibody (Abcam) was added directly to the TBS-T solution for 2 h at room temperature. PVDF membranes were washed again and incubated with a 1 : 1000 of horseradish peroxidase-conjugated antirabbit IgG Envision+ (Dako Japan Inc., Kyoto, Japan) as a secondary antibody for 20 min at room temperature. Finally, the membranes were incubated with ECL+-horseradish peroxidase substrate solution included in the ECL+kit (Amersham Biosciences UK Ltd, UK), and immunoblotting was visualised by exposing the membrane to Hyperfilm (Amersham) (Endo *et al*, 2004; Kasamatsu *et al*, 2005).

### Immunohistochemistry

Immunohistochemical staining was performed on 4-*μ*m sections of paraffin-embedded specimens with the use of rabbit anti-human stathmin polyclonal antibody (Abcam). Briefly, after deparaffinisation and hydration, the slides were treated with endogenous peroxidase in 0.3% H_2_O_2_ for 30 min, after which the sections were blocked for 2 h at room temperature with 1.5% blocking serum (Santa Cruz Biotechnology, Santa Cruz, CA, USA) in PBS before reacting with antistathmin antibody (1 : 1000 dilution) at room temperature in a moist chamber for overnight. Upon incubation with the primary antibody, the specimens were washed three times in phosphate-buffered saline and treated with Envision reagent (Dako) followed by colour development in 3,3′-diaminobenzidine tetrahydrochloride (Dako). Finally, the slides were lightly counterstained with haematoxylin, dehydrated with ethanol, cleaned with xylene, and mounted. As a negative control, duplicate sections were immunostained without exposure to primary antibodies. To quantitate the state of stathmin protein expression, the mean percentage of positive tumour cells was determined in at least five random fields at × 400 magnification in each section. The intensity of the stathmin-immunoreaction was scored as follows: 1+, weak; 2+, moderate; and 3+, intense. The percentage of positive tumour cells and the staining intensity then were multiplied to produce a stathmin-immunohistochemical staining score (Tanaka *et al*, 2003; Shimada *et al*, 2005). Cases with a stathmin score grater than 86.16 (the highest score for normal tissue) were defined as positive. These judgments were made by two independent pathologists, neither of whom had knowledge or information pertaining to the patients' clinical status. Statistical significance was evaluated by the Fisher's exact test or the Mann–Whitney *U*-test. *P*<0.05 was considered significant.

### mRNA expression analysis

Among the OSCC cases studied by immunohistochemistry, expression levels of *stathmin* mRNA were examined in 20 patients with OSCC (10 stathmin-negative and 10 stathmin-positive cases) from whom RNA was available from primary tumours and from paired specimens of normal oral tissue. Control reactions were prepared in parallel without reverse transcriptase. In addition, expression levels of *stathmin* mRNA were examined in OSCC-derived cell lines (HSC-2, HSC-3, HSC-4, Ca9-22, OK92, and Sa3) and HNOKs. Real-time quantitative reverse transcriptase–polymerase chain reaction (qRT–PCR) was performed with a single method using a LightCycler FastStart DNA Master SYBR Green 1 Kit (Roche Diagnostics GmbH, Mannheim, Germany), according to the procedure provided by the manufacturer. The primer sequences used to analyse *stathmin* mRNA expression were forward 5′-CCCCTTTCCCCTCCAAAGAA-3′ and reverse 5′-TCGCAAACGTTCCAGTTTGG-3′. The sequence of specific primers was checked before use to avoid amplification of genomic DNA or pseudogenes by Primer3 program (available at http://www-genome.wi.mit.edu/cgi-bin/primer/primer3_www.cgi). The PCR reactions using LightCycler (Roche) apparatus were carried out in a final volume of 20 *μ*l of a reaction mixture consisting of 2 *μ*l of FirstStart DNA Master SYBR Green I mix (Roche), 3 mM MgCl_2_, and 0.2 *μ*l of the primers, according to the manufacturer's instructions. The reaction mixture was then loaded into glass capillary tubes and subjected to an initial denaturation at 95°C for 10 min, followed by 45 rounds of amplification at 95°C (10 s) for denaturation, 63°C (10 s) for annealing, and 72°C for extension, with a temperature slope of 20°C s^−1^, performed in the LightCycler. The transcript amount for the *stathmin* gene was estimated from the respective standard curves and normalised to the glyceraldehyde-3-phosphate dehydrogenase (GAPDH) (forward 5′-CATCTCTGCCCCCTCTGCTGA-3′ and reverse 5′-GGATGACCTTGCCCACAGCCT-3′) transcript amount determined in corresponding samples. The statistical significance of the gene expression levels between stathmin-positive and stathmin-negative cases was calculated with the Mann–Whitney *U*-test. *P*<0.05 was considered significant. Data are expressed as the means (±s.d.) of two independent experiments with triplicate samples.

### Statistical analysis

The overall survival time was defined as the interval between the date of treatment and the date of death or until the last objective follow-up information was obtained. Disease-free survival time was regarded as the time interval between tumour treatment and detection of the first locoregional recurrence and/or distant metastasis or the date of last follow-up, whichever occurred first. Disease-free survival or overall survival according to stathmin overexpression was constructed using Kaplan–Meier method. The comparison among curves was performed by the log-rank test with 95% of significance.

## RESULTS

### Analyses of protein expression of stathmin in OSCC-derived cell lines

**Figure 1:**
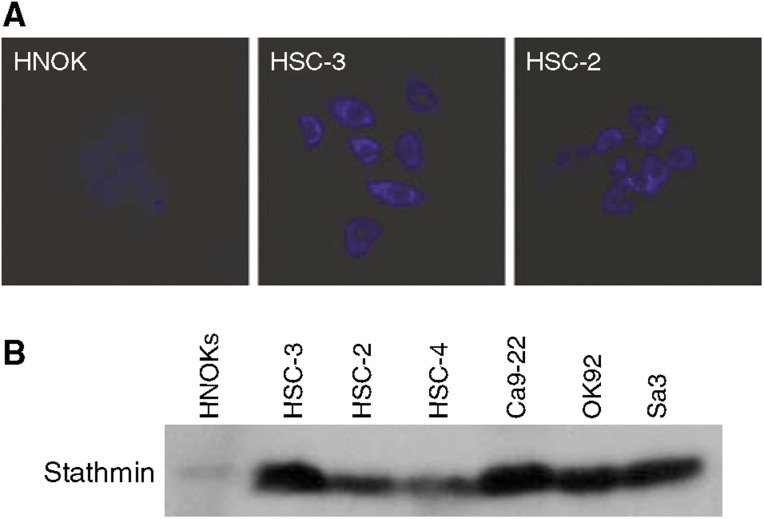
Representative results of expression of stathmin protein in OSCC-derived cell lines. (**A**) Immunocytochemical analysis shows strong immunoreactivity of stathmin in OSCC-derived cell lines (HSC-2 and HSC-3) compared with the HNOKs. (**B**) Western blot analysis of stathmin protein in OSCC-derived cell lines and HNOKs. HNOKs extracts do not significantly express stathmin protein. OSCC-derived-cell lines extracts exhibiting stathmin protein expression.

We previously showed the upregulation of stathmin in OSCC-derived cell lines (HSC-3 and HSC-2) (Koike *et al*, 2005). In the current study, we further assessed the level of stathmin protein expression in OSCC-derived cell lines (HSC-3 and HSC-2) and HNOKs by immnofluorescence analysis. Figure 1A shows typical results of immnofluorescence. Strong immunoreactivity of stathmin protein was detected in the cytoplasm of OSCC-derived cell lines compared with the HNOKs. We also evaluated the state of stathmin protein expression in four additional OSCC-derived cell lines (HSC-4, ca9–22, OK92, and Sa3) by Western blot analysis. Figure 1B shows representative results. The size of the band was detected as 17 kDa, as reported by Feuerstein and Cooper (1983), and a significant increase in stathmin expression was observed in all OSCC-derived cell lines compared with the HNOKs. Immnofluorescence and Western blotting experiments confirmed the results from 2-D gel analysis and protein identification by mass spectrometry.

### Immunohistochemistry

**Figure 2:**
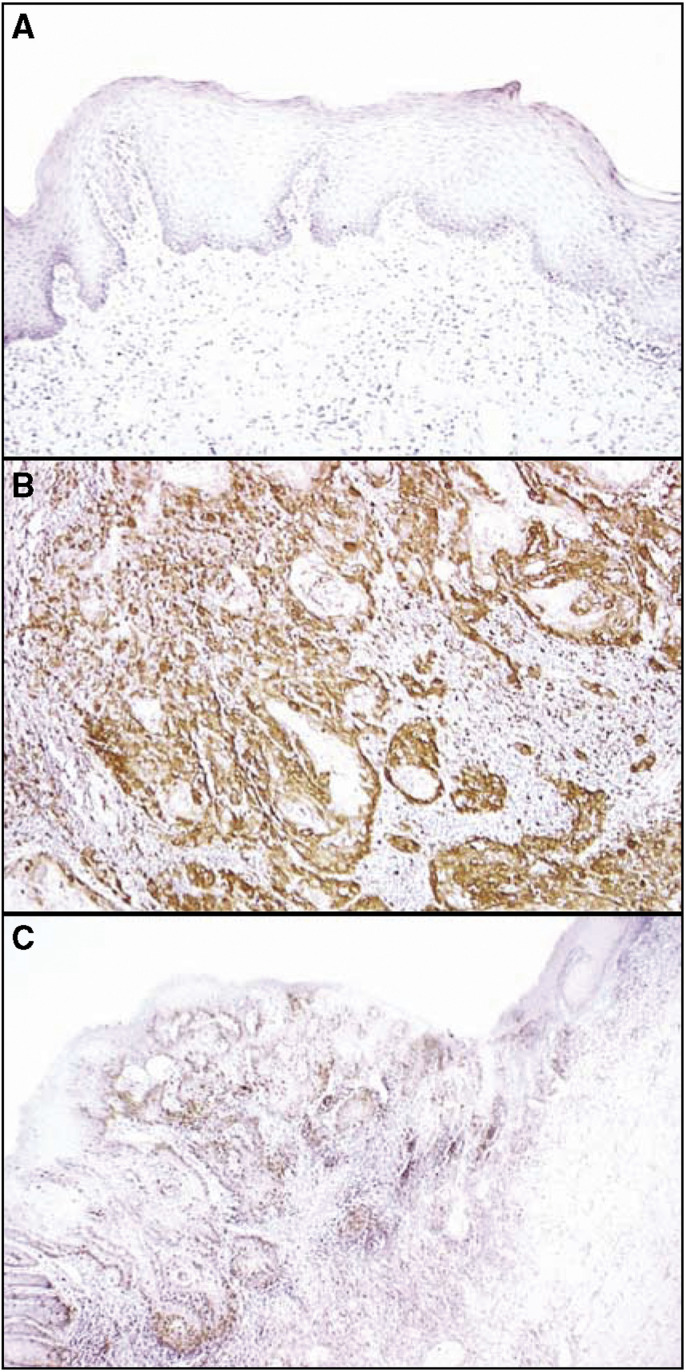
Immunohistochemical staining of stathmin in normal and primary OSCCs. (**A**) Normal oral tissue shows no stathmin protein expression. Original magnification, × 200. (**B**) Stathmin-positive case of OSCC. Strong positive immunoreaction for stathmin is detected in the cytoplasm. Original magnification, × 200. (**C**) The border between normal epithelium (right side) and the dysplastic lesion (left side) is seen. While no stathmin expression is detected in normal epithelial cellular cytoplasm, strong stathmin protein expression is evident in the lesion. Original magnification, × 100.

**Figure 3:**
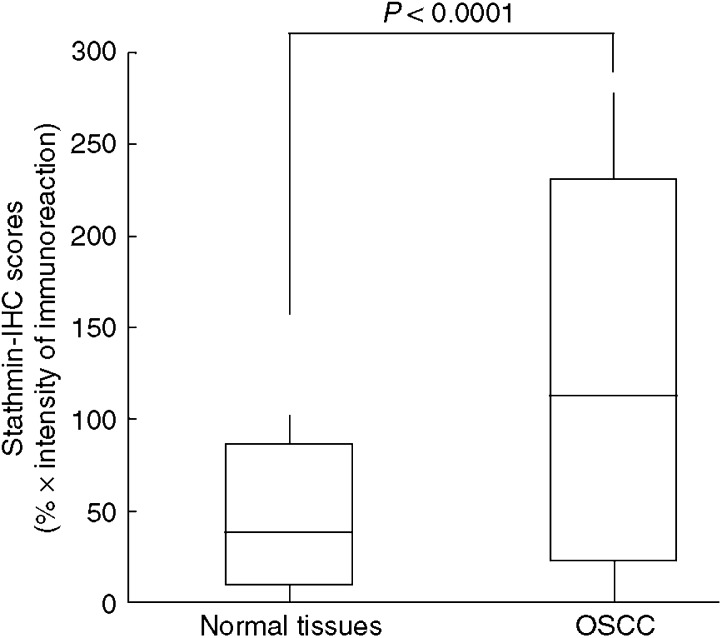
State of stathmin protein expression in normal tissues (*n*=81) and primary OSCCs (*n*=81). The stathmin-immunohistochemistry scores are calculated as follows: stathmin-immunohistochemistry score=(% of positive tumour cells) × the staining intensity. Stathmin protein expression in OSCCs is significantly higher than in normal oral tissues (*P*<0.0001, Mann–Whitney *U*-test). The results represent the mean±s.d. IHC, immunohistochemistry.

**Figure 4:**
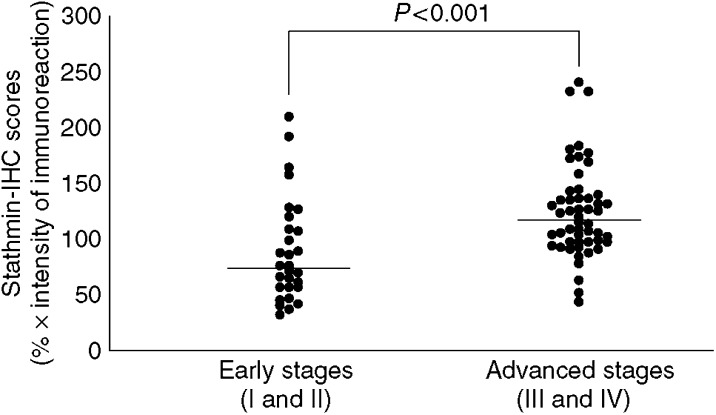
State of stathmin protein expression in patients with early-stage (I and II) OSCC (*n*=29) and patients with advanced stages (III and IV) OSCC (*n*=52). The stathmin-immunohistochemistry scores are calculated as follows: stathmin-immunohistochemistry score=(% of positive tumour cells) × the staining intensity. Stathmin protein expression in advanced stages is significantly higher than in the early stages (*P*<0.001, Mann–Whitney *U*-test). The results represent the mean. IHC, immunohistochemistry.

A total of 81 patients with OSCC were identified for whom there was adequate histologic material available for immunohistochemical analysis. The correlation between the clinicopathologic characteristics of the patients with OSCC and the status of stathmin expression is summarised in Table 1. Normal oral mucosa specimens had no or significant downregulation of stathmin expression and were considered stathmin-negative (Fisher's exact test). Among the tumours examined, 53 of 81 cases (65%) had a stathmin immunoreaction in the cytoplasm of the tumour cells (Table 1). Moreover, expression of these proteins was correlated with the TNM stage grading of OSCCs (*P*=0.035; Table 1). Representative results for stathmin protein expression in normal oral tissue and primary OSCCs are shown in Figure 2. The stathmin-immunohistochemistry scores for normal tissues and OSCCs ranged from 8.77 to 86.16 (mean, 32.59) and 21.83 to 231.0 (mean, 101.6), respectively. The stathmin expression levels in primary OSCCs were significantly higher than those in normal oral tissues (Mann–Whitney *U*-test, *P*<0.0001; Figure 3). Furthermore, the stathmin-immunohistochemistry scores for early stages (I and II) and advanced stages (III and IV) ranged from 21.83 to 199.4 (mean, 80.56) and 32.6 to 230.9 (mean, 113.1), respectively. The stathmin expression levels were significantly higher in the OSCC group with advanced stage disease compared with the group with early stage disease (Mann–Whitney *U*-test, *P*<0.001; Figure 4).

### mRNA expression analysis

**Figure 5:**
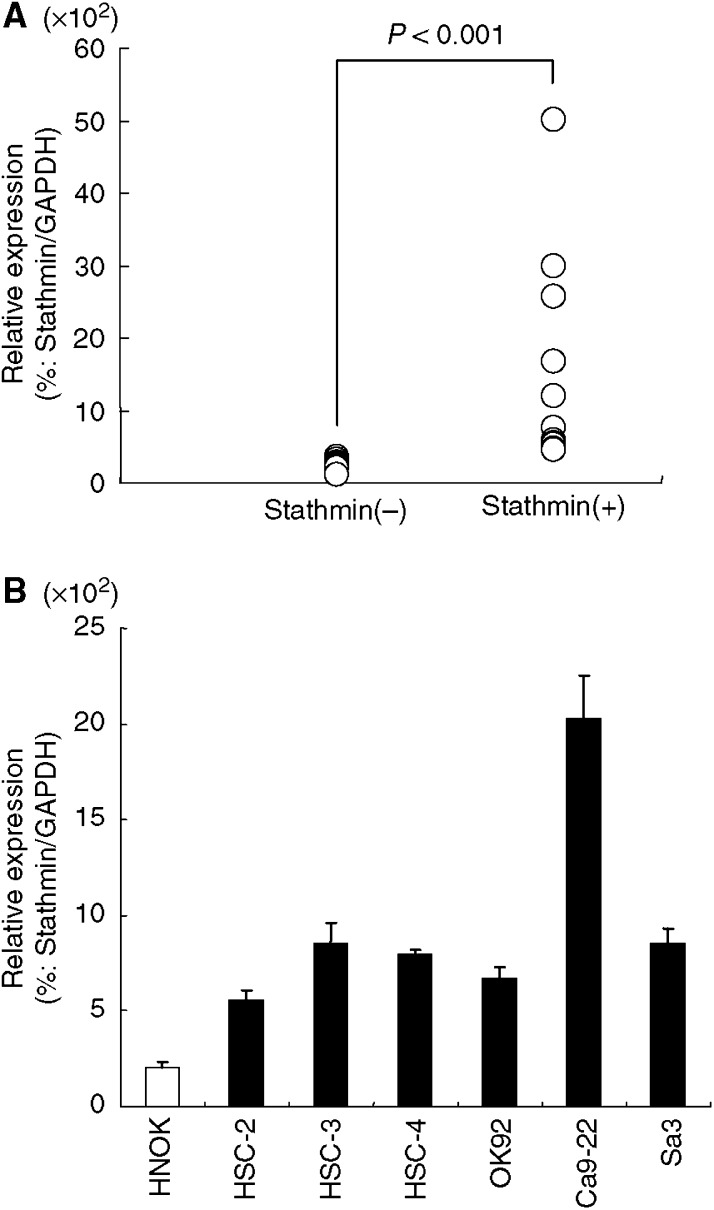
*Stathmin* mRNA expression status in primary OSCCs and OSCC-derived cell lines. (**A**) Comparison of *stathmin* mRNA expression levels between stathmin-positive and stathmin-negative cases classified by immunohistochemistry analysis. There is a significant difference in the *stathmin* mRNA expression levels between negative and positive cases (*P*<0.001, Mann–Whitney *U*-test). (**B**) Quantification of mRNA levels in OSCC-derived cell lines by qRT–PCR analysis. Significant upregulation of the *stathmin* gene is seen in all OSCC-derived cell lines compared to *stathmin* mRNA expression in HNOKs. Data are expressed as means±s.d.

qRT–PCR analysis data were matched to protein expression levels studied by immunohistochemistry scores. The *stathmin* mRNA expression levels were significantly increased in primary tumours of randomly selected stathmin-positive cases (*n*=10) compared with randomly selected stathmin-negative cases (*n*=10, Mann–Whitney *U*-test, *P*<0.001; Figure 5A). Relative mRNA expression levels in negative and positive cases ranged from 11 to 35.6 (mean, 22.5) and 48 to 500.3 (mean, 163.7), respectively. As shown in Figure 5B, a significant increase in the expression of *stathmin* mRNA occurred in all OSCC-derived cell lines examined compared with the HNOKs. The *stathmin* mRNA expression levels for the HNOKs and OSCC-derived cell lines ranged from 15.1 to 23.1 (mean, 19.4) and 56 to 200.3 (mean, 95.8), respectively. Therefore, *stathmin* mRNA expression levels were consistent with the protein expression status.

### Prognostic significance of stathmin expression in OSCC

**Figure 6:**
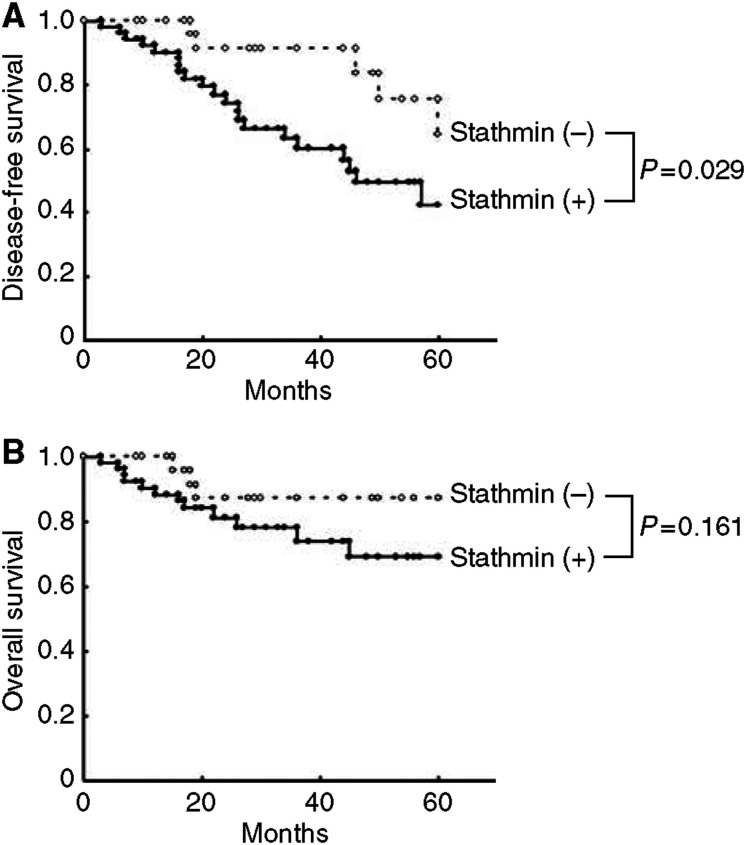
Kaplan–Meier curves for disease-free survival (**A**) or overall survival (**B**) according to stathmin overexpression in patients with OSCC (log-rank test, **A**, *P*=0.029; **B**, *P*=0.161). When stathmin overexpression was observed, survival rate was not significant (**B**), but disease rate increased significantly (**A**).

The disease-free survival curves in relation to stathmin overexpression are shown in Figure 6A. Stathmin overexpression was found to be a significant for disease-free survival by the log-rank survival analysis (*P*=0.029). The overall survival curves in relation to stathmin overexpression are shown in Figure 6B. Although stathmin overexpression is poorer than that of the stathmin negative expression, no significant difference was found (*P*=0.161). Because the length of follow-up ranged from 3 to 60 months, 5-year survival could not be yet available.

## DISCUSSION

Because the functional molecules in cells are protein, proteome analysis based on 2-D gel electrophoresis is believed to have several advantages over cDNA/oligonucleotide microarray systems for clinical use. Proteomic studies of clinical tumour samples have led to the identification of cancer-specific protein markers, which provide a basis for developing new methods for early diagnosis and early detection and clues to understanding the molecular characterisation of cancer progression (Emmert-Buck *et al*, 2000; Hanash, 2000; Liotta and Petricoin, 2000; Prasannan *et al*, 2000). Previously, we reported that a significant increase in stathmin protein expression was observed in OSCC-derived cell lines, using a 2-D-DIGE system and MALDI-TOF/MS (Koike *et al*, 2005).

Stathmin was first identified as a 17-kDa cytosolic protein that is rapidly phosphorylated when HL60 leukemic cells undergo terminal differentiation and cease to proliferate (Feuerstein and Cooper, 1983). In addition, stathmin is a conserved cytosolic protein that has been studied in various cellular systems such as oncoprotein 18, prosolin, p19, 19K, p18, and op18 (Belmont and Mitchison, 1996; Laird and Shalloway, 1997). Recent studies have reported that this gene plays a critical role in mitosis and possibly other cellular processes (Rubin and Atweh, 2004). Therefore, stathmin has attracted the attention of many investigators because of its high level of expression in many types of cancer, including leukemia and lymphoma (Hanash *et al*, 1988; Luo *et al*, 1991; Ghosh *et al*, 1993; Roos *et al*, 1993), prostate carcinoma (Friedrich *et al*, 1995), ovarian carcinoma (Price *et al*, 2000), Wilms' tumour (Takahashi *et al*, 2002), breast carcinoma (Bieche *et al*, 1998), and adenoid cystic carcinoma of the salivary glands (Nakashima *et al*, 2005). Recent studies have reported that stathmin is highly expressed in more advanced stages of the disease in Wilms' tumour (Takahashi *et al*, 2002). In addition, high protein expression of stathmin correlated with a poor prognosis in breast cancer (Brattsand, 2000). However, the status of stathmin in OSCC remains unclear. Thus, we selected stathmin for further investigation.

To clarify the relative status of stathmin in OSCC, we investigated the protein/mRNA expression in a series of OSCC-derived cell lines and human primary OSCCs using immunofluorescence, Western blot analysis, qRT–PCR, and immunohistochemistry. Significant increases in stathmin protein and mRNA expression levels were observed in the OSCC-derived cell lines examined compared with the HNOKs. We also detected a comparatively strong tumour cell-localised cytoplasmic stathmin-immunoreaction in primary OSCCs. By evaluating the stathmin immunohistochemistry scores using the Mann–Whitney *U*-test, significant stathmin upregulation was evident in the primary OSCCs (*P*<0.0001, OSCC *vs* corresponding normal tissues) compared with normal tissues. qRT–PCR analysis data were matched to protein expression levels studied by Western blot analysis and immunohistochemistry. The stathmin protein expression levels in primary OSCCs were significantly associated with TNM stage grading (*P*=0.035). Moreover, the state of the protein stathmin expression differed significantly between the early stages (I and II) and the advanced stages (III and IV) (Mann–Whitney *U*-test, *P*<0.001). Our results suggest a significant association between stathmin and the clinical stages of tumours, with upregulation of stathmin in the advanced stages (III and IV) of OSCC, which may result in tumour aggressiveness and the progression of OSCC. Moreover, this study has shown that the stathmin overexpression is closely related to the disease-free survival (*P*=0.029), suggesting that stathmin could be a novel prognostic/therapeutic marker.

Recent studies have shown that inhibition of stathmin expression in malignant cells interferes with their orderly progression through the cell cycle and abrogates their transformed phenotype (Mistry and Atweh, 2002). Thus, stathmin provides an attractive molecular target for disrupting the mitotic apparatus and arresting the growth of malignant cells. In addition, several studies have shown that stathmin is a potential therapeutic target because a *stathmin*-transfected lung cancer cell line has increased sensitivity to *Vinca* alkaloids (Nishio *et al*, 2001), and antisense inhibition of *stathmin* expression showed a synergistic apoptotic effect in combination with paclitaxel treatment (Iancu *et al*, 2000). Further studies with a larger population will improve our ability to treat this cancer.

Based on our data, we have concluded that stathmin is not only frequently overexpressed in OSCC, but also may play an important role in the progression and patient prognosis of this disease. In addition, the differential expression status of stathmin between the early and advanced stages of OSCC may provide insights into the process of tumorigenicity and for planning new treatment strategies.

## Figures and Tables

**Table 1:** Correlation between the expression of Stathmin and clinical classification in OSCCs

		**Immunostaining results (no. of patients (%))**		
**Clinical classification**	**Total**	**Stathmin (−)**	**Stathmin (+)**	***P*-value^a^**
*Age at surgery (years)*				
<60	22	8 (36)	14 (64)	
60 ⩽, <70	26	6 (23)	20 (77)	0.654
70⩽	33	14 (42)	19 (58)	
				
*Gender*				
Male	48	16 (33)	32 (67)	0.961
Female	33	12 (36)	21 (64)	
				
*T-primary tumour*				
T1	10	7 (70)	3 (30)	
T2	39	15 (38)	24 (62)	0.149
T3	13	2 (15)	11 (85)	
T4	19	4 (21)	15 (79)	
				
*N-regional lymph node*				
N(−)	41	18 (44)	23 (56)	0.202
N(+)	40	10 (25)	30 (75)	
				
*Stage*				
I	8	6 (75)	2 (25)	
II	21	11 (52)	10 (48)	0.035
III	13	4 (31)	9 (69)	
IV	39	7 (18)	32 (72)	
				
*Histopathologic type*				
Well differentiated	51	17 (33)	34 (67)	
Moderately differentiated	22	8 (36)	14 (64)	0.999
Poorly differentiated	8	3 (38)	5 (62)	
				
*Tumour site*				
Gingiva	27	6 (22)	21 (78)	
Tongue	30	15 (50)	15 (50)	0.997
Buccal mocosa	7	2 (29)	5 (71)	
Oral floor	10	3 (30)	7 (70)	
Oropharynx	7	1 (14)	6 (86)	

a A provability of *P*<0.05 was defined as significant.

## References

[bib1] Belmont LD, Mitchison TJ (1996) Identification of a protein that interacts with tubulin dimers and increases the catastrophe rate of microtubules. Cell 84: 623–631 8598048 10.1016/s0092-8674(00)81037-5

[bib2] Bieche I, Lachkar S, Becette V, Cifuentes-Diaz C, Sobel A, Lidereau R, Curmi PA (1998) Overexpression of the stathmin gene in a subset of human breast cancer. Br J Cancer 78: 701–719 9743287 10.1038/bjc.1998.565 PMC2062973

[bib3] Brattsand G (2000) Correlation of oncoprotein 18/stathmin expression in human breast cancer with established prognostic factors. Br J Cancer 83: 311–318 10917544 10.1054/bjoc.2000.1264 PMC2374559

[bib4] Charlwood J, Skehel JM, Camilleri P (2001) Analysis of N-linked oligosaccharides released from glycoproteins separated by two-dimensional gel electrophoresis. Anal Biochem 284: 49–59 10.1006/abio.2000.4687 10933855

[bib5] Emmert-Buck MR, Gillespie JW, Paweletz CP, Ornstein DK, Basrur V, Appella E, Wang QH, Huang J, Hu N, Taylor P, Petricoin EF (2000) An approach to proteomic analysis of human tumors. Mol Carcinog 27: 158–165 10708477

[bib6] Endo Y, Uzawa K, Mochida Y, Shiiba M, Bukawa H, Yokoe H, Tanzawa H (2004) Sarcoendoplasmic reticulum Ca(2+) ATPase type 2 downregulated in human oral squamous-cell carcinoma. Int J Cancer 110: 225–231 15069686 10.1002/ijc.20118

[bib7] Feuerstein N, Cooper HL (1983) Rapid phosphorylation induced by phorbol ester in HL-60 cells. J Biol Chem 258: 10786–10793 6577004

[bib8] Friedrich B, Gronberg H, Landstrom M, Gullberg M, Bergh A (1995) Differentiation stage specific expression of oncoprotein 18 in human and rat prostatic adenocarcinoma. Prostate 27: 102–109 7638082 10.1002/pros.2990270207

[bib9] Ghosh PK, Anderson J, Cohen N, Takeshita K, Atweh GF, Lebowitz P (1993) Expression of the leukemia-associated gene, p18, in normal and malignant tissues: inactivation of expression in a patient with cleaved B cell lymphoma/leukemia. Oncogene 8: 2869–2872 8397372

[bib10] Hanash SM (2000) Biomedical applications of two-dimensional electrophoresis using immobilized pH gradients: current status. Electrophoresis 21: 1202–1209 10786892 10.1002/(SICI)1522-2683(20000401)21:6<1202::AID-ELPS1202>3.0.CO;2-I

[bib11] Hanash SM, Strahler JR, Kuick R, Chu EHY, Nichols D (1988) Identification of a polypeptide associated with the malignant phenotype in acute leukemia. J Biol Chem 263: 12813–12815 3417633

[bib12] Iancu C, Mistry SJ, Arkin S, Atweh GF (2000) Taxol and anti-stathmin therapy: a synergistic combination that targets the mitotic spindle. Cancer Res 60: 3537–3541 10910066

[bib13] Kasamatsu A, Uzawa K, Nakashima D, Koike H, Shiiba M, Bukawa H, Yokoe H, Tanzawa H (2005) Galectin-9 as a regulator of cellular adhesion in human oral squamous-cell carcinoma cell lines. Int J Mol Med 16: 269–273 16012760

[bib14] Koike H, Uzawa K, Nakashima D, Shimada K, Kato Y, Higo M, Kouzu Y, Endo Y, Kasamatsu A, Tanzawa H (2005) Identification of differentially expressed proteins in oral squamous-cell carcinoma using a global proteomic approach. Int J Oncol 27: 59–67 15942644

[bib15] Laird AD, Shalloway D (1997) Oncoprotein signalling and mitosis. Cell Signal 9: 249–255 9218124 10.1016/s0898-6568(96)00176-3

[bib16] Larsson T, Bergstrom J, Nilsson C, Karlsson KA (2000) Use of an affinity proteomics approach for the identification of low-abundant bacterial adhesionsas applied on the Lewis (b)-binding adhesin of *Helicobacterpylori*. FEBS Lett 469: 155–158 10713262 10.1016/s0014-5793(00)01270-9

[bib17] Liotta L, Petricoin E (2000) Molecular profiling of human cancer. Nat Rev Genet 1: 48–56 11262874 10.1038/35049567

[bib18] Lippman SM, Sudbo J, Hong WK (2005) Oral cancer prevention and the evolution of molecular-targeted drug development. J Clin Oncol 23: 346–356 15637397 10.1200/JCO.2005.09.128

[bib19] Luo X-N, Arcasoy MO, Brickner HE, Mistry S, Schechter AD, Atweh GF (1991) Regulated expression of p18, a major phosphoprotein of leukemic cells. J Biol Chem 266: 21004–21010 1939149

[bib20] Mistry SJ, Atweh GF (2002) Role of stathmin in the regulation of the mitotic spindle: potential applications in cancer therapy. Mt Sinai J Med 69: 299–304 12415323

[bib21] Nakashima D, Uzawa K, Kasamatsu A, Koike H, Endo Y, Saito K, Hashitani S, Numata T, Urade M, Tanzawa H (2005) Protein expression profiling identifies maspin and stathmin as potential biomarkers of adenoid cystic carcinoma of the salivary glands. Int J Cancer 118: 704–713 10.1002/ijc.21318 16094606

[bib22] Nishio K, Nakamura T, Koh Y, Kanzawa F, Tamura T, Saijo N (2001) Oncoprotein 18 overexpression increases the sensitivity to vindesine in the human lung carcinoma cells. Cancer 91: 1494–1499 11301397 10.1002/1097-0142(20010415)91:8<1494::aid-cncr1157>3.0.co;2-7

[bib23] Pandey A, Mann M (2000) Proteomics to study genes and genomes. Nature 405: 837–846 10866210 10.1038/35015709

[bib24] Prasannan L, Misek DE, Hinderer R, Michon J, Geiger JD, Hanash SM (2000) Identification of *β*-tubulin isoforms as tumor antigens in neuroblastoma. Clin Cancer Res 6: 3949–3956 11051243

[bib25] Price DK, Ball JR, Bahrani-Mostafavi Z, Vachris JC, Kaufman JS, Naumann RW, Higgins RV, Hall JB (2000) The phosphoprotein Op18/stathmin is differentially expressed in ovarian cancer. Cancer Invest 18: 722–730 11107442 10.3109/07357900009012204

[bib26] Roos G, Brattsand G, Landberg G, Marklund U, Gullberg M (1993) Expression of oncoprotein 18 in human leukemias and lymphomas. Leukemia 7: 1538–1546 8412315

[bib27] Rubin CI, Atweh GF (2004) The role of stathmin in the regulation of the cell cycle. J Cell Biochem 93: 242–250 15368352 10.1002/jcb.20187

[bib28] Rudert F (2000) Genomics and proteomics tools for the clinic. Curr Opin Mol Ther 2: 633–642 11249740

[bib29] Shimada K, Uzawa K, Kato M, Endo Y, Shiiba M, Bukawa H, Yokoe H, Seki N, Tanzawa H (2005) Aberrant expression of RAB1A in human tongue cancer. Br J Cancer 92: 1915–1921 15870709 10.1038/sj.bjc.6602594 PMC2361773

[bib30] Takahashi K, Kanazawa H, Akiyama Y, Tazaki S, Takahara M, Muto T, Sato K (1989) Establishment and characterization of a cell line (SAS) from poorly differentiated human squamous-cell carcinoma of the tongue. J Jpn Stomatol Soc 38: 20–28

[bib31] Takahashi M, Yang XJ, Lavery TT, Furge KA, Williams BO, Tretiakova M, Montag A, Vogelzang NJ, Re GG, Garvin AJ, Soderhall S, Kagawa S, Hazel-Martin D, Nordenskjold A, Teh BT (2002) Gene expression profiling of favorable histology Wilms tumors and its correlation with clinical features. Cancer Res 62: 6598–6605 12438255

[bib32] Tanaka C, Uzawa K, Shibahara T, Yokoe H, Noma H, Tanzawa H (2003) Expression of an inhibitor of apoptosis, survivin, in oral carcinogenesis. J Dent Res 82: 607–611 12885844 10.1177/154405910308200807

[bib33] Wilkins MR, Sanchez JC, Gooley AA, Appel RD, Humphery-Smith L, Hochstrasser DF, Williams KL (1996) Progress with proteome projects: why all proteins expressed by a genome should be identified and how to do it. Biotechnol Gen Eng Rev 13: 19–50 10.1080/02648725.1996.10647923 8948108

